# Loneliness, Food Practices, and Ageing: The Inclusion of Qualitative Evidence in Health Promotion Programmes

**DOI:** 10.3390/nu17172906

**Published:** 2025-09-08

**Authors:** Elena Freire Paz, Borja Rivero Jiménez, David Conde Caballero, Lorenzo Mariano Juárez

**Affiliations:** 1 Department of Philosophy and Anthropology, University of Santiago de Compostela, 15705 Santiago de Compostela, Spain; elena.freire@usc.es; 2 Department of Nursing, University of Extremadura, 10003 Cáceres, Spain; dcondecab@unex.es; 3 Department of Psychology and Anthropology, University of Extremadura, 10003 Cáceres, Spain; lorenmariano@unex.es

**Keywords:** loneliness, social isolation, food practices, ageing, qualitative evidence

## Abstract

**Background/Objectives**: Social sciences have described the 21st century as the ‘era of loneliness’, a time marked by the subjective and often distressing experience of lacking meaningful social connections. While loneliness has been linked to poorer health outcomes, its relationship with dietary practices remains underexplored. **Methods**: This ethnographic study examines the impact of loneliness on the eating habits of older adults, based on fieldwork conducted in Galicia and Extremadura (Spain) between November 2024 and April 2025. Using semi-structured interviews (*n* = 25) and participant observation in domestic settings, we examined everyday food practices. Data were examined using thematic analysis. **Results**: Participants acknowledged the connection between diet and health, although their dietary practices often failed to meet nutritional recommendations. Loneliness and social isolation influenced food choices and cooking routines, often leading to less nutritious diets. These effects were shaped by gender and social class. Many participants associated the decline of shared meals and food-related sociability with a diminished quality of life. The absence of a meaningful companion was experienced as a profound loss, prompting various coping strategies. **Conclusions**: The findings highlight that food holds both nutritional and symbolic value. Health promotion strategies should address the social and emotional dimensions of eating. Loneliness disrupts food practices at multiple levels, calling for public health responses that integrate biological, cultural, and care perspectives.

## 1. Introduction

The 21st century has been defined as ‘the lonely century’ [1]. Loneliness is an issue attracting increasing attention and concern, often described as a ‘pandemic’ or ‘epidemic’ [2,3]. In the first official European study on loneliness, 13% of older adults reported feeling lonely ‘most’ or ‘all of the time’ in the previous four weeks, while up to 35% said they felt lonely ‘at least sometimes’ during that period [4]. Available data revealed disparities between Northern European countries and those in the South and East, with a higher incidence of loneliness reported in the latter regions [5,6]. In Spain, loneliness is often incorrectly associated with the number of single-person households, mistakenly equating loneliness with the act of living alone. However, living alone does not necessarily lead to feelings of loneliness or social isolation. Indeed, many individuals living alone maintain strong social connections and report low levels of loneliness [7,8].

To provide an inclusive term that integrates both the subjective experience of loneliness and the objective measure of social isolation, recent scientific literature—particularly in English-speaking countries—has started using the concept of ‘Social Isolation and Loneliness’ (SIL) [9,10,11]. In a recent national survey, conducted in 2024, a significant portion of the population reported experiencing loneliness: 20% of all adults, including 14.5% of those aged 65–74 years and 20% of those aged 75 years and over [12]. In Galicia, approximately one in five individuals experiences unwanted loneliness (19.8%), with chronic loneliness affecting 15.2% of the population, above the national average of 13.5% [13]. For Extremadura, no region-specific studies are currently available.

The causes of this increase in loneliness are multifaceted. At the individual level, the literature identifies several factors associated with loneliness, including age (being over 65 years old) [5,14], living alone [15,16], being female [17,18], and having a chronic illness [19]. Recent approaches have highlighted the role of social determinants, introducing the ‘lonelygenic environments’ conceptual model [20]. This concept encompasses factors that hinder the creation of social bonds, such as the rise of remote work, heavy social media use—which can deepen disconnection rather than foster connection—and the increasing influence of exclusionary issues, including poverty, racism, and xenophobia. Additionally, a pervasive modern trend toward individualism further erodes the sense of belonging.

Biomedical research has demonstrated that loneliness can both precede and be a consequence of various health conditions. As such, it has been compared to other major health risk factors, including smoking, obesity, and high cholesterol [21,22]. Loneliness or social isolation has been linked to a 14% increased risk of all-cause mortality, with a particularly significant impact on mental health. Social isolation alone may raise the risk of death by up to 32%, particularly affecting cognitive and physical health [23]. Other studies have pointed to an increased risk of myocardial infarction and coronary heart disease [24], higher blood pressure [25], and a general decline in overall health [26,27]. Prolonged loneliness generates both physiological and psychological stress that may lead to metabolic alterations, including changes in appetite, weight regulation, and systemic inflammation. It also increases susceptibility to late-life depression and may accelerate cognitive decline, thereby increasing the risk of dementia. Social isolation has also been linked to acute psychological stress [28], Alzheimer’s disease [29], depression [30], and cognitive decline [31].

Studies addressing the relationship between loneliness, dietary habits, and health risks are relatively scarce. Some researchers suggest that reduced commensality can influence decisions about how food is acquired, prepared, served, consumed, or even stored [32]. Eating alone is often perceived as less enjoyable [33,34], which may reduce motivation to cook or eat, and increase the likelihood of skipping meals or making poorer food choices [35,36]. Understanding and addressing this reality requires insights from the social sciences, which have the capacity to reveal the symbolic meanings behind food ideologies. Nutritional issues are complex and multifaceted, extending beyond physical and physiological factors to include social and cultural dimensions that play a crucial role in shaping beliefs about food consumption [37].

This study aims to deepen understanding of the relationship between loneliness, diet, and health in older adults, drawing on social science perspectives and methodologies. Specifically, it seeks to achieve the following:

(a) Examine the key role of commensality—or, in many cases, its absence—in eating habits and food ideologies, highlighting how this may affect not only the quality and regularity of food intake but also the meaning people assign to eating and its overall influence on their health and quality of life, real and perceived;
(b) Highlight the importance of companionship and shared meals for the quality of life of older adults, while also examining their role in promoting healthier eating habits;
(c) Suggest that nutritional health promotion programmes consider the available qualitative evidence and acknowledge the significance of cultural and social factors in dietary choices.

## 2. Materials and Methods

This qualitative study is grounded in the classic premises and methods of ethnographic fieldwork, employing an inductive–deductive approach.

### 2.1. Participants and Settings

Fieldwork was conducted in several locations in the Autonomous Communities of Extremadura and Galicia (Spain). Although this study forms part of a broader research project conducted across multiple regions of the country, this article focuses on two regions, which were selected for their similar levels of ageing, rurality, and prevalence of single-person households relative to other regions.

We used purposive sampling for sample selection, prioritising older adults living alone. A total of 25 individuals participated in individual, in-depth interviews (Table 1). Inclusion criteria required participants to be aged 65 years or older, live independently, and demonstrate sufficient cognitive and mental capacity to engage in the interview. Exclusion criteria were being under 65 years, diagnosed with dementia or pre-dementia, or living with others. All participants provided written informed consent. Before the interviews, participants completed the Social and Emotional Loneliness Scale for Adults (SELSA-S) [38], which had been adapted into Spanish [39].

The interviews focused on dietary practices and food ideologies among this age group, complemented by observations conducted in food supply locations and participants’ domestic environments, which served as observational units within the methodological triangulation. Additionally, to avoid bias in equating loneliness with living alone, interviews were also conducted with older adults residing with others (these were not included in the main sample). All interviews were audio-recorded. The total duration of the interview recordings was 24 h and 37 min, with the duration of individual interviews ranging from 33 min (shortest) to 72 min (longest). Among the participants, 80% (*n* = 20) were female and 20% (*n* = 5) were male. Their mean age was 76.48 years. To ensure confidentiality, all participants are referred to by pseudonyms throughout the article.

### 2.2. Data Collection

Data collection took place between October 2024 and May 2025, employing different techniques. Informal conversations with older adults provided a preliminary approach and guidance in developing the interview categories [40,41]. These informal conversations took place in various contexts within the locations listed in Table 1. Field diary notes included information about the interviews and descriptions of the observational units [42,43].

The interview guide design (Table 2) outlined subject areas and theoretical questions, informed by a critical review of the related literature. This guide was applied flexibly, adapting it in each interview according to the social rapport established with the participants. Pilot interviews were conducted to evaluate the relevance and clarity of the guide. Additionally, the interviewers followed an inductive approach, maintaining a flexible and receptive attitude towards incorporating emerging topics not initially considered [44]. The interviews were conducted by researchers with recognised expertise in qualitative methodologies and extensive experience in both research and teaching.

### 2.3. Ethics

All participants provided informed consent by signing a document that explained the study’s purpose, research objectives, and their right to withdraw from the study at any time if they so wished. Participant confidentiality was guaranteed throughout the entire research process. The first author anonymised the participants’ data, and the rest of the research team worked exclusively with the coded dataset. The project received approval from the Bioethics and Biosafety Committee of the University of Extremadura (176/2024). The research complied with current Spanish legislation on personal data protection (Organic Law 3/2018 of 5 December, on Personal Data Protection and Guarantee of Digital Rights). The study adhered to the ethical principles outlined in the Declaration of Helsinki and the Belmont Report.

### 2.4. Data Analysis

All interviews were audio-recorded and accompanied by field notes taken during the sessions. Data collection was conducted by a team of three researchers with extensive experience in qualitative methods and in-depth interviewing. All interviews were transcribed verbatim, with contextual observations from field notes included when pertinent. To protect confidentiality, all personal details that could potentially identify participants were removed. We used ATLAS.ti qualitative analysis software (Scientific Software Development GmbH, Berlin, Germany, version 7.5.7 for Windows) to analyse the interview transcripts.

The interpretative process was guided by the core premises of thematic analysis [45]. Categories were identified and analysed through systematic content coding: (1) preliminary units of analysis were established following an initial reading of the transcripts; and (2) using the constant comparison method, openly coded data were grouped into concepts, from which categories were subsequently generated. We initially defined 37 categories, which were then grouped into 11. Once data had been disaggregated into categories and subcategories, their relationships were systematically examined through axial coding [46]. Finally, after a selective coding process, the categories were grouped into three main themes. One member of the research team coded the initial categories of analysis using an inductive–deductive approach [47,48]. Two researchers independently examined, analysed, and reinterpreted the interviews, while a third researcher resolved any discrepancies, thereby completing the triangulation.

## 3. Results

Three key themes emerged from the interview data: the central role of health in shaping food ideologies during ageing; the influence of loneliness on the food choices and dietary habits of older adults; and the importance of preserving or pursuing the social dimension of food consumption, even in contexts of loneliness. Table 3 contains a summary of the main results.

### 3.1. The Role of Health in Shaping Food Ideologies During Ageing

The interviews revealed that older adults associated their diets with health preservation, including food choices, preparation methods, and portion sizes. From a discursive perspective, health emerged as a dominant cultural value, with food choices and eating habits positioned as central components of individual self-care strategies:

‘[My diet] is very simple; I don’t eat anything too complicated. Everything is either pan-fried or microwaved. [...] I can’t anymore, my stomach just can’t handle greasy foods; they don’t agree with me. [...] everything is just plain: boiled, steamed, pan-fried.’

(F73E)

‘I don’t eat meat very often now–for instance, beef or anything like that. I’ll have chicken or lamb on rare occasions, but I mainly eat vegetables, more than anything else. Yes, more vegetables than anything else. Vegetables, and fruit, and fish, more or less... All types, really. But I tend to go for salmon quite a lot–you know, because of the omega.’

(F73G)

Most narratives reflected alignment with primary care professionals’ recommendations, linking food choices to prevention and treatment strategies, while still allowing for a certain degree of enjoyment and pleasure:

‘I eat whatever I feel like, but also what is good for my health–I usually go for fish, you know, oily fish, because it’s good for cholesterol.’

(F73G)

The primary health-related concept was the idea of ‘eating little and well’–often accompanied by limiting food intake at the end of the day. This practice, a natural form of intermittent fasting, was described in the participants’ accounts as contributing to good health:

‘Around six [...] and a half, yes, and after dinner I do not eat anything solid–usually, before bed, I’ll have one of those relaxing herbal teas, a placebo. That’s my routine.’

(M71G)

The narratives emerging from the interviews highlighted self-care and health as central factors shaping their food choices. However, some participants acknowledged that their daily routines did not always match these ideals. Additionally, their definition of healthy food and healthy eating practices sometimes incorporated peculiar viewpoints, such as including cured meats or high-calorie foods in their diets, e.g., ‘a hearty cocido [traditional Spanish one-pot stew] with pork belly.’ Participants described these foods as healthy, using adjectives such as ‘traditional,’ ‘natural,’ ‘good quality,’ or ‘from our land,’ which reflects a culturally diverse understanding of what constitutes healthy eating from a nutritional perspective [49]. The theoretical significance attributed to health was often overshadowed by the value placed on hedonistic desires or cravings, which were justified through an ambivalent logic: ageing is perceived both as a stage requiring self-care and as one warranting the pursuit of pleasure. Food and eating are framed as having a dual role, as both nourishment and medicine, but also as a source of enjoyment.

‘Chocolate truffles really call to me at night. Chocolate truffles at night. Before going to bed, I have to eat some chocolate. I need something sweet, and that sweet thing is chocolate. I almost always have a truffle or a piece of chocolate nougat, or something similar, but it’s almost always a truffle. [...] I remember once–someone was giving us a talk about all that, and I said: “Look, you can take anything away from me. You can tell me all I need to eat, a healthy diet, that kind of diet. But the little square... the little square of chocolate I have before bed at night–that’s medicine to me.”’

(F72G)

It is important to acknowledge the role of gender in shaping discourses and practices related to care and nutrition. Female participants appeared to place greater importance on self-care and health, emphasising moderation in food consumption and managing body weight. Male participants, on the other hand, while sharing concerns about weight control as a sign of wellbeing, tended to associate health with moderation in behaviours such as alcohol consumption and choosing ‘good quality’ foods.

‘I have lost six kilos since I stopped drinking wine and alcohol [...] I asked [my GP], “Can I have a sip of wine just to get rid of the taste of food? So I can actually taste the wine?” And he said, “If it’s just a sip at lunch and a sip with your evening meal, that’s OK.” Just a sip while I eat. I make sure to eat and wait, wait, and then, when I finish the last spoonful, I’ll have a sip.’

(M85E)

Material conditions, including socioeconomic class and social status, also inform perceptions of what constitutes health. This is especially significant considering that ageing is a well-known factor contributing to economic vulnerability, a particularly acute issue in the settings targeted by our research. In our interviews, tensions emerged between the theoretical discourses and the reality of what was financially attainable:

‘Fish is very expensive: I eat frozen haddock, the one that comes in blocks like little cakes, and I alternate between fish and meat once a week. [...] I no longer get any treats: if something is expensive, I only buy it once.’

(F73E)

‘Those little cans of tuna that come in packs of three. Of course. No, [I know] I have to buy it, but I mean, my goodness, it’s so expensive. But yes, I’ll have to buy it, I’ll have to open a can of sardines one of these nights–but I think it costs so much money, when cans used to be the cheapest thing.’

(F93E)

In summary, the participants’ accounts suggested that they understood the crucial role of dietary choices in maintaining health. However, healthcare professionals must be aware of the contradictions and social determinants that shape actual dietary practices—such as the role of pleasure, culturally rooted perceptions of what constitute healthy foods, gender norms, and socioeconomic status—and how these might undermine health-promoting discourses.

### 3.2. The Influence of Loneliness on the Food Ideologies, Food Choices, and Dietary Habits of Older Adults

In addition to these tensions, participants’ narratives highlighted the impact of loneliness on food choices and dietary practices in older adults. Significant changes in food acquisition habits were noted, with several participants reporting a reduction in the purchase of fresh products alongside an increased reliance on items with a longer shelf life. In some cases, food acquisition practices were shaped by ideologies related to food waste. For instance, fruit consumption tended to decline among individuals living alone, as the increased risk of spoilage led them to purchase smaller quantities or stop buying fruit altogether to avoid waste Similarly, participants increasingly turned to ready-made meals or pre-prepared foods rather than cooking from scratch, motivated by concerns over food waste, as exemplified by the traditional cold tomato soup gazpacho, widely available nowadays in ready-to-serve formats. Particularly among men, although not exclusively, an increased tendency to purchase and consume more canned foods was noted:

‘Well, I buy cans of cooked chickpeas or lentils. Or those cans with grilled pork–I just reheat [the content] and it’s ready. Because, as I said, I can’t remember anything, I don’t feel up to anything.’

(F78E)

As mentioned previously, gender plays a significant role in these contexts of loneliness. Regarding food preparation practices, female participants possess cultural expertise derived from their traditional cooking responsibilities within the family unit, resulting in a greater capacity to adapt to changing circumstances. Interviews revealed that they drew upon a broader range of resources, grounded in experience and practical knowledge. This made them more adept in diverse preparation methods, not only in terms of cooking techniques, which were often similar (e.g., boiling), but also in their inventive and resourceful use of ingredients.

‘Look, I’ve boiled some Swiss chard, that’s food for today and food for tomorrow. Now, at midday, I add a potato, and I can have chard with a potato. Tomorrow I’ll add some dried beans, those yellow ones I like so much [...] from El Barco of Ávila. I got two kilos. I’ve prepared some beans, I’ve got some Swiss chard, and I’m eating like a queen.’

(F78E)

Changes in meal frequency, food selection, cooking techniques, and the underlying motivations for these choices were also observed. For example, many female participants, once relieved of family responsibilities in old age, chose to step away from the decades-long routine obligation of preparing meals. In such cases, participants expressed that maintaining previous levels of effort was no longer worthwhile, resulting in simplified culinary routines, reduced meal variety, and a reliance on quicker, more practical food options. There was a prevailing perception that cooking for oneself required less effort than preparing meals for others. While this attitude was observed among some female participants, it was more common among male participants. Male interviewees exhibited more uniform and repetitive eating patterns, with less diversity in ingredients and preparation methods, despite expressing interest in maintaining good health at this life stage.

‘Potatoes with ribs, or ribs in gravy. Chicken... roasted–hearty food. Because I used to eat a lot. [...] No, I don’t do that anymore–not even occasionally, never. You do not do these kinds of things anymore. [...] I don’t feel like cooking it or eating it [...] No, now that I’m on my own, I just don’t do that. I eat well. I don’t get complicated with chorizo sausage or... No, on my own, I don’t do that.’

(F83E)

Changes in the eating habits of older adults living alone also included their food preservation strategies. In contrast to the traditional health benefits attributed to consuming fresh produce, a growing trend towards normalising the use of frozen food was noticed, motivated both by the difficulty of calculating individual portions and by convenience. In contexts where there is no longer anyone to share meals with, freezing food becomes a practical and efficient solution. Some participants stressed the importance of avoiding food waste, a value deeply ingrained in this generation [36]. However, this could occasionally lead to lapses in food safety assurance: ‘Sometimes you don’t get the amount right and you end up eating it the next day or the day after–you’re not going just to throw it away.’ From a practical perspective, maintaining frozen food supplies provided a reliable means of ensuring food availability when needed.

‘Look, what I cooked today will last me for three days. Today I’ll eat this, and tomorrow I’ll have some soup–because I took some out of the freezer. I thought, you know, for dinner or whenever, I just took it out. Because I didn’t have anything else. And now, look what I have.’

(F93E)

Dietary practices should also consider the experiential and qualitative dimensions of mealtimes, particularly in relation to commensality. In this context, culinary loneliness reflects an acknowledged sense of loss often associated with a perceived decline in quality of life. Eating alone frequently becomes a melancholic and monotonous experience, where the absence of companionship is profoundly felt. As several interviewees highlighted, feelings of loneliness were most acute during mealtimes:

‘We always used to eat together, my wife and I–I remember how it was back then–there was conversation, there was life. But now... I just sit at the table, put my plate down, and eat quickly, without really enjoying it. Often, I don’t even bother with a tablecloth. Eating alone is really hard–really sad. I just cook whatever, enough to take the edge off feeling hungry–but it is not the same. The silence is the worst part–when you’re eating alone, that’s the hardest thing.’

(M85E)

In some cases, the experience of loneliness led to simplified dietary practices. As mentioned above, in contexts of loneliness, mealtimes tend to become simpler, with the gradual disappearance of elements that once imbued them with social and cultural significance. This issue also revealed significant gender-related differences. On the one hand, female participants tended to simplify their preparation methods, which were often adapted to healthy cooking ideologies, such as pan-fried meat and fish, boiled vegetables, or simple vegetable salads. A low level of culinary complexity characterises these dishes. Some also resorted to less healthy, yet equally simple, options, such as a glass of milk with biscuits or the so-called ‘pan migao’ [bread crumbled into warm milk]. For male participants, on the other hand, meal simplification often involved increased reliance on canned foods, as well as the frequent consumption of cured meats with bread, occasionally accompanied by simple additions such as fried eggs with ham or chorizo. Male participants also reported eating out more frequently when financially feasible. It is also worth noting that male participants appeared more receptive to consuming prepared meals, such as those provided by social services through various meal delivery programs. However, female participants tended to reject these types of meals, expressing feelings of embarrassment or perceiving their use as an acknowledgement of personal inadequacy or dependency.

‘I’m more of a salad and pan-fried something person […] Some nights I don’t eat anything other than an apple and a yoghurt. Or strawberries and yoghurt. Or a banana [...]. Fruit and a yoghurt. Almost always.’

(F83E)

‘Sometimes I feel a bit lazy [...] so I just grab whatever there is and that’s it. [...] Like, a bit of chorizo, some cheese, a bit of patatera sausage–which I love–I just spread it [on bread].’

(F78E)

Loneliness was also associated with the elimination of bread, a historically and culturally central component of the Spanish diet. This does not merely reflect a change in taste or habit, but a profound sense of loss. In a prior study, we observed that for many individuals, eating without bread was perceived as nearly equivalent to not eating at all [49]. Bread, a fundamental food symbol—particularly for older generations—signifies far more than mere sustenance: it represents companionship, daily ritual, and communal sharing. Among our participants, bread was sometimes excluded from the diet. In other cases, it was replaced by industrially produced sliced bread, which has a longer shelf life, thereby reducing the need for daily purchasing. When there is no one to share it with, the regular practice of purchasing freshly baked bread every morning becomes redundant and potentially wasteful.

‘No, not at all. I haven’t eaten bread in a long time. I just don’t feel like it. These days I’ll just buy some sliced bread and eat a slice or two–not even every day.’

(M78E)

The experience of loneliness has a significant impact on food choices and practices among older adults, often leading to changes in their eating habits. From food acquisition to meal preparation, the overall process tends to become simplified when there are no companions with whom to share meals. Consequently, loneliness often results in the adoption of simplified food choices and, in some cases, the complete abandonment of cooking. The appeal of complex, nutritionally rich dishes appears to be sustained only when they fulfil a social role, i.e., social interaction and sharing.

### 3.3. Food Consumption as a Social Necessity: The Importance of Preserving or Pursuing the Social Dimension of Food Consumption, Even in Contexts of Loneliness

Our participants’ accounts revealed that, even in contexts marked by loneliness, eating was conceived as a fundamentally social act with meanings that extended beyond its purely nutritional function. The combined experience of ageing and loneliness adversely affected the motivation to prepare, consume, and enjoy food.

Eating alone was often experienced as a meaningless act. While some participants acknowledged that loneliness could afford a certain degree of dietary agency—such as the freedom to decide what to cook or when to eat—it could also lead to food losing its significance. Several participants described their eating experiences as merely ‘eating for the sake of eating’ or ‘eating out of duty,’ suggesting a pervasive sense of loss and a lack of motivation. Eating alone becomes an experience that triggers memories and evokes a profound sense of nostalgia for past meals. The dining table, once a daily gathering place for the family, becomes a space where their absence is most acutely experienced. For some of our participants, mealtimes were among the loneliest moments in their daily routines. Food served as a poignant reminder of the past commensality, when every dish was shared with others. When lacking an audience to appreciate their efforts, some female participants—traditionally responsible for preparing food in their households—reported diminished motivation. For them, cooking and eating as a family were not merely functional activities but symbols of everyday life that had been supplanted by their opposite–loneliness. Cooking and eating served as fundamental social practices that connected individuals and shaped their identities as social beings. Consequently, loneliness often diminished the significance and meaning attributed to these activities.

‘Because my mother’s up there, in the village–if it was just for me, half the time I wouldn’t even cook... I eat there every day, although sometimes I don’t–It just depends on how I feel when I wake up. If I don’t feel like it, I don’t eat there, I don’t. If I don’t go one day, I’ll go there the next–but generally I don’t feel very motivated. It’s all just out of duty. Every bit of it. If it weren’t for my mother, if I were on my own, some days I wouldn’t cook at all. No. I’d eat whatever, because... Anything but cooking. Living on your own is… Maybe it’s fine for some people, but it doesn’t work for me. When you’re on your own–I overthink, there is too much going on inside my head.’

(F67G)

This diminished interest in cooking and eating often resulted in reduced dietary variety, less time devoted to meal preparation, and a decline in the care taken with food in general. Nonetheless, within this context, a variety of cultural coping strategies could be observed. The mediated presence of television or radio often serves as a surrogate for social interaction, filling domestic spaces with sound and providing a form of symbolic companionship.

‘Yes, the TV, and this thing [mobile] for gossip too.’

(F93E)

‘I put the TV on; I always put it on. Always, when I’m eating. Look, because it brings me a sense of peace. I know what we’ll see. Let me explain it to you. I know it is a box full of lies. Yes, but... I’m aware that for every true thing, there are two hundred lies. But... It brings me peace. It brings me peace.’

(F78E)

Once again, gender-based differences emerged from the interviews. Male participants demonstrated a significantly greater tendency to cope by frequenting bars, small eateries, or restaurants offering daily set menus, particularly for their main meal of the day. These settings foster the development of informal social networks, even in the absence of explicit interaction, recreating relationships of trust and familiarity, much like those found in domestic environments. The repetitive nature of these encounters allows for kitchen and dining staff to become familiar with each guest’s tastes, preferences, and specific dietary requirements, developing a form of daily sociability structured by routine.

‘Usually, I eat out about half the time, like at least two days a week. Uh... I almost always eat with the same people. On weekends, I usually eat out–mostly on Saturdays and Sundays. There are usually three of us. I also have a friend, and we’d go to another village to eat. I know this lady who’s a really good cook.’

(M76G)

Although some female participants also engaged in these types of practices, they did so differently, typically arranging to go with others in advance and seldom visiting bars or cafes alone. In their narratives, this practice was framed as an occasional necessity rather than a routine behaviour, frequently accompanied by a sense of discomfort or reluctance towards these environments.

‘Eating alone in a restaurant... It’s not for me. Only when really necessary. If you know what I mean–Sometimes when I’m out and don’t have time to go home or whatever, I’ll go to a restaurant. But I would just grab a sandwich and eat it on a bench instead. How could I eat alone, in a restaurant?’

(F73G)

‘On Sundays, we go out for a bit. I might have a small beer. A small beer or two, depending. For an aperitif, around midday. Otherwise, the mid-morning coffee. But only if Ana is going to be there, otherwise I won’t go. Last week I went out for lunch with my friends, but...’

(F81G)

These narratives suggest that food consumption during ageing still transcends mere biological necessity, retaining a strong social and symbolic dimension, even in contexts of loneliness. Our participants’ accounts illustrated how older adults actively managed, negotiated, and adapted their daily eating practices in response to the emotional and social circumstances shaping their lives. Even within contexts of loneliness, the social significance of eating persisted through efforts to maintain ritualised and orderly practices, including adherence to mealtimes, the careful arrangement of the table with a tablecloth—even when dining alone—and the use of particular utensils designated for each occasion. These everyday routines and gestures served as symbolic mechanisms that evoked the continuity of a past social order, functioning as acts of resistance against the fragmentation of social and familial bonds in later life.

‘I realised that if I left a cup on the–on the table, and didn’t tidy it up–it would still be there the next day, and that was frightening, you know. I mean, little things that made me see–this is what loneliness really feels like. So, the next step was–I think people our age need some kind of incentive, and along with that, a goal. To show we’re no less capable than anyone else. An incentive and a goal that challenges us, but without overwhelming us [...] The first thing I did was set a schedule for myself. I mean, you need to–to have a routine. I kept getting up at the same time every day, eating at the same time, having dinner, keeping track of my spending–everything, just like I used to do with my husband. Only now, I was doing it alone.’

(F73E)

Particularly among female participants, meticulous attention to household details seemed to acquire a heightened symbolic value in structuring everyday routines. The use of embroidered tablecloths and cross- or back-stitched kitchen towels, and the careful storage of dry goods in neatly labelled containers are not just practical concerns: they are practices that contribute to the symbolic ordering of the domestic space, and, by extension, the individual’s lived experience. Similarly, special tableware, glassware, coffee sets, and cutlery sets were carefully preserved. While no longer routinely used, these items were maintained in excellent condition and continued to hold a prominent place within the domestic space, particularly in dining rooms. Beyond their material value, these objects represented a legacy inherited from mothers and grandmothers. Their enduring symbolic and affective significance evoked key moments in the interviewees’ life histories, such as marriage or when they started their own family. Consequently, they provided continuity and meaning to personal narratives in a present shaped by loneliness.

‘My husband’s family was very, very, very... So, I had to use a little tablecloth. I’d often say, “No, the glass is clean.” But still, the little tablecloth. So, they are still there, in a drawer–two little tablecloths. For this little table [...] To dress it up.’

(F93E)

‘Oh! I always use tablecloths, individual ones, but a tablecloth always. You see, my husband, without one... And my daughters would say, “But Dad, paper napkins are better.” Oh, no! They had to be proper cloth ones. So, I had all these proper tablecloths, and I would bring out both cloth and paper napkins, so everyone could choose. And, afterwards–I really like them, I’ve always had [...] I still keep the same traditions as when my husband was with us.’

(F85G)

The emergence of loneliness resulted in the erosion of traditional eating habits and a perceived decline in the quality of life. These factors appeared to contribute to the adoption of less nutritious dietary practices and food choices. However, our research also revealed performative behaviours seeking to maintain sociability despite these challenges. Individually, the ‘companionship’ offered by television, the anticipation of Sundays when children return home for a family meal, seeking socialisation in shared spaces such as bars or cafes, and efforts to imbue mealtimes with meaning, e.g., the use of special tablecloths and tableware as mnemonic devices to prevent memories from fading away, are relevant factors that should be acknowledged and incorporated into health promotion programmes.

## 4. Discussion

### 4.1. Tensions Between Self-Care and Pleasure in the Eating Habits of Older Adults

Increased life expectancy in industrialised countries presents a range of challenges, especially in enhancing public health and ensuring quality of life for older adults [50,51]. Food ideologies, nutritional knowledge, and dietary practices play a critical role in achieving these objectives. The design of health promotion strategies should ideally be informed by theoretical frameworks that incorporate notions of cultural change and its impact on health-related behaviours [52,53]. However, social and cultural factors are not always recognised as key health determinants within nutritional interventions. In-depth interviews conducted in this study reveal that loneliness and social isolation have a significant impact on the eating habits of older adults. While health remains a central value, old age is marked by tension: fear of illness and mortality drives self-care strategies, yet moments of pleasure and enjoyment of the present also hold significance. These dualities should be acknowledged and integrated into health promotion programmes.

Our analysis of participants’ accounts reveals that health awareness plays a central role in their lives. Participants demonstrate understanding of the connections between eating habits and self-care, which is consistent with findings from other studies [54,55,56]. However, their everyday practices are characterised by a tension between what they recognise as healthy and what is subjectively appealing, often resulting in resistance to health professionals’ recommendations, as observed, for instance, with alcohol consumption [57]. It has long been recognised that while nutritional knowledge is essential, it is not enough to drive changes in consumer eating behaviour [58]. Although health holds a theoretically dominant status, its principles often struggle with the strong social influence of established eating habits. This tension between health guidelines and subjective experience has been documented in contexts of vulnerability [49] and, in the present study, it emerges as a symbolic expression of agency: choosing certain foods against the advice of healthcare professionals serves as an act of identity affirmation and an exercise of control over the present. One participant exemplified this dynamic by regularly consuming fried eggs several nights a week despite medical advice to the contrary. This behaviour reflected resistance to the medicalisation of old age and asserted the legitimacy of everyday pleasures. It also suggested the importance of avoiding overly restrictive evidence-based approaches to healthcare, as some less beneficial social behaviours create spaces for pleasure that significantly enhance quality of life.

### 4.2. Impact of Loneliness on Eating Habits

Scientific research has highlighted the negative impact of loneliness on morbidity and mortality rates [23]. Based on our research, loneliness affects eating habits in different ways, including a reduced interest in food acquisition, preparation, and consumption, as well as decreased satisfaction with mealtimes, ultimately leading to a diminished perception of personal quality of life. There is consistent evidence that dietary patterns are closely intertwined with the quality of life of older adults [59], potentially playing a role in the broader context of declining health in later life. Health promotion interventions should also be informed by approaches that consider gender as a central category and a key determinant of health [60,61]. Our study demonstrates that the impact of loneliness on eating habits is gender-dependent, a factor that should be considered in the design of nutritional health policies and interventions. For some women, experiences of sudden loneliness—such as widowhood—may open up new possibilities for empowerment and control that were previously limited or unavailable [62]. However, our findings also underscore a loss of meaning and identity as caregiving roles—including the act of preparing food for others—diminish or disappear. This sometimes results in reduced attention to dietary practices, leading to nutritionally poor choices. Female participants described engaging in ongoing self-care through dietary restraint, particularly at the end of the day. They also favoured raw, boiled, or pan-fried foods, and relied on products such as dairy, fruits, and eggs, reflecting a common value of frugality. However, they also recognised the necessity of deriving pleasure through the consumption of sweet, sugary foods, e.g., milk with crumbled biscuits at dinnertime. In the case of male participants, loneliness was associated with a decline in eating habits. Many interviewees demonstrated limited knowledge of food preparation (‘I don’t even know how to fry an egg’), and frequently relied on cold, plain meals consisting of canned food, cured meats, and fruit. Representations of ageing masculinity highlight moderation and self-control as positive markers of personal responsibility and wellbeing [7]. This might suggest reduced consumption; however, actual intake often remained substantial. Our research demonstrates the direct impact of social isolation on food choices, preparation, and consumption. In many instances, diets were marked by limited variety and quality, featuring quick, frugal meals composed of eggs, soups, or cured meats, along with reduced consumption of fresh products. As Devine [63] and Rozin [64] suggest, food choices are dynamic processes shaped by social experiences, cultural meanings, and individual biographies. Similarly, many of our participants redefined their eating habits in response to loneliness by prioritising functionality over nutritional or symbolic considerations.

Health promotion interventions based on the simplistic ‘promote healthy habits, inspire change’ framework often overlook important gender-related factors–such as varying levels of participation in programs like ‘Meals on Wheels’ or the use of canteens and soup kitchens, as well as class-related issues: while loneliness impacts individuals differently, the capacity to access and navigate available resources is strongly influenced by social position and income [65]. Our findings reinforce and expand existing evidence on the influence of social and emotional factors on food choices throughout a person’s lifespan [63], providing valuable insights to inform more effective health promotion strategies aimed at ageing populations.

### 4.3. Social and Cultural Dimensions of Eating Alone

Although much research on ageing, health, and nutrition focuses on dietary approaches and the identification of deficiency states [66,67], we argue that social and cultural variables are a crucial factor that often receives insufficient attention. This study highlights the value of ethnographic methods in providing critical insights into the multifaceted complexities of loneliness and its impact on health outcomes. The anthropology of food and eating has explored how sociality and reciprocity are produced and sustained through food choices and practices [68]. Culturally shaped eating practices serve as expressions of identity, helping to define what it means to be a person and what constitutes a life worth living [69]. Indeed, a significant part of our worldview—and our perceived quality of life—is rooted in the acts of eating and tasting [70]. Illness or therapeutic treatments that impair the senses of taste and smell have been described as diminishing quality of life [71], to the extent that, for some individuals, life is no longer seen as worth living. In the Spanish context, the perceived connections between food, identity, and commensality have historically been so strong [72] that eating alone was, until recently, associated with notions of animality or savagery. Consequently, recognising and addressing loneliness as a complex issue is essential in the design of healthcare interventions. Eating alone—the loss of commensality—diminishes the social and symbolic meanings of food, reducing it to mere sustenance [73], a perception that has tangible effects on both actual and perceived health. Emphasising the social and cultural dimensions of food consumption would broaden both the scope and effectiveness of nutritional interventions.

The experiential and qualitative dimensions of food consumption shape dietary practices. In this context, culinary loneliness is often accompanied by a recognition of loss that is closely associated with diminished perceived quality of life [74]. The quality and enjoyment of food eaten alone evoke a sense of absence and incompleteness, making the food less flavourful and the experience less satisfying. It is not merely a diminished interest in food, but also an acknowledgement that loneliness disrupts life by eroding key social spaces essential to making life worth living. Our research revealed certain practices aimed at restoring these social and symbolic dimensions, underscoring their importance and the need for health professionals to acknowledge rather than overlook them. For instance, while some studies associate eating while watching television with increased obesity rates [75], our participants describe it as a cultural practice of simulated companionship that lends meaning to the act of eating. This may indicate certain potential as both a tool and an effective medium for health promotion interventions [76]. Similarly, recognising the need to eat in ‘meaningful’ company should guide care home policies to allow individuals to dine with family and friends, rather than restricting them to eating only with fellow residents.

## 5. Limitations and Practical Implications

### 5.1. Limitations

Although the chosen methodological approach allows for an interpretive understanding of the phenomenon, it is important to acknowledge certain limitations. Regarding the size and composition of the sample, although discursive saturation was achieved, it is possible that the sample may not capture sufficient diversity of perspectives. A certain degree of selection bias may also be present, potentially influenced by the researchers’ network of contacts and participant accessibility.

Additionally, the cognitive status of participants was assessed subjectively. For future studies, it would be advisable to incorporate quantitative data using standardised instruments such as the Montreal Cognitive Assessment (MoCA). Socioeconomic status was also assessed subjectively, although most participants reported limited incomes, primarily derived from retirement or widowhood pensions. Future research should address this variable in a more systematic manner, which could improve sample selection criteria and enhance analytical outcomes. Furthermore, no specific assessment of participants’ health status was conducted; however, future research could explore individuals with particular physical or psychological health conditions.

Although a triangulation process was employed during interpretation the influence of the researchers’ theoretical frameworks cannot be entirely eliminated. In the Spanish context, loneliness sometimes associated with the stigma of abandonment, which can give rise to processes of social desirability and discursive reconstruction that should be acknowledged. Finally, the study examines the impact of loneliness on eating practices through interviews with participants in specific life contexts (e.g., duration of loneliness, levels of acceptance, diverse causes), which may act as potential confounding factors. Replicating this research in other regional contexts is, therefore, necessary.

### 5.2. Practical Implications

Our findings underscore the importance of integrating social and emotional dimensions into health promotion programmes targeting older adults. This study provides qualitative evidence on how loneliness and social isolation influence food choices, affecting quality of life and potentially increasing dependency. This approach aligns with intervention methods based on social prescribing, which use social and community resources and activities as complements or alternatives to traditional biomedical treatments to enhance patients’ health and wellbeing [77,78]. Incorporating this approach would not only improve dietary habits, but also reinforce social networks and a sense of belonging among vulnerable populations. This perspective is relevant given studies that highlight the importance of lifestyles where food sociability plays a central and beneficial role—such as the Mediterranean diet [79]—and could thus constitute a potential strategy for addressing conditions like depression and, in this case, loneliness.

It is also essential to consider calls for a more effective integration of health and social interventions, as proposed by Garattini et al. [80] and Schoemakers et al. [11]. These authors noted inadequate inter-sectoral coordination and the lack of standardised evaluation frameworks for interventions targeting loneliness in older adults. In this regard, qualitative evidence—such as that generated in this study—offers critical insight into the contextual and cultural specificities that shape experiences of loneliness and eating, providing an essential foundation for designing equitable and culturally sensitive interventions [81].

## 6. Conclusions

Loneliness and social isolation among older adults may lead to changes in eating habits, resulting in poorer dietary choices regarding both food selection and preparation methods. These changes, mediated by factors such as gender and social class, may also contribute to the health decline associated with ageing and loneliness.

Loneliness disrupts daily life and challenges key cultural values associated with food consumption, such as sociability and pleasure. This not only contributes to the development of less balanced eating habits, but it is also experienced as a profound loss, negatively affecting the perceived quality of life of older adults.

Nutrition-focused health promotion programmes must place greater emphasis on the social and cultural dimensions of eating habits. Shortcomings within the ‘food culture’ are as significant as biological and nutritional deficiencies, and effective interventions should address these issues at both the individual and collective levels.

## Figures and Tables

**Table 1:** Socio-demographic profile of the participants.

Name	Age	Gender	Location
Teresa	83	Female	Extremadura
Ana Belén	81	Female	Galicia
Javier	76	Male	Galicia
Ramón	85	Male	Extremadura
Laura	77	Female	Galicia
Andrés	71	Male	Galicia
Nati	72	Female	Galicia
Elena	78	Female	Extremadura
Julia	73	Female	Galicia
Rosa M.	73	Female	Galicia
Eugenia	93	Female	Extremadura
Mercedes	79	Female	Galicia
Alicia	73	Female	Extremadura
Loli	71	Female	Galicia
Aurora	66	Female	Galicia
Inés	78	Female	Extremadura
Berta	69	Female	Galicia
Noelia	70	Female	Galicia
Amalia	78	Female	Extremadura
Silvia	66	Female	Galicia
Hugo	75	Male	Extremadura
Nuria	84	Female	Galicia
Celia	85	Female	Galicia
Soledad	78	Female	Extremadura
Luis	78	Male	Galicia

**Table 2:** Interview guide.

Blocks/Theoretical Questions	Guide Questions
Identification	Do you live alone? Do you feel isolated? Do you feel lonely?
Food acquisition practices past and present	Have you noticed any changes in how you buy food compared to the past? What are your food-buying habits now that you live alone? Do you buy food more or less often than you used to? Do you still go to the same types of shops as before?
Food preparation	How do you prepare food these days? Are there any kitchen utensils or methods you use now that you didn’t use previously? Are there any kitchen utensils or methods that you have stopped using? Are there any dishes that you no longer prepare? Is there a reason why? Are there any new dishes you’ve started preparing? If so, why? How often do you eat pre-prepared food? Has this always been the case? Is there a reason why?
Mealtimes	Do you eat at the same time every day? Would you say you eat faster or more slowly than you used to? How long does it usually take to eat your meals? Do you ever skip any meals? Why do you eat when you eat?
Eating habits	Would you say you eat more or less than you used to? Do you usually have both a first and second course, or just one dish? Do you often watch TV, listen to the radio, browse the internet, or read during meals? Where do you usually eat your meals? Has this changed over time?
Table setting	Do you typically set the table with items such as a tablecloth, various tableware, or cutlery? Do you use the same tableware as before? How many pieces of tableware do you normally use? Are there any items of tableware that you no longer use? Visual ethnography of tableware.
Emotional and symbolic aspects	Do you usually eat whatever you feel like, or do you try to follow dietary recommendations–for instance, from your doctor? When you follow dietary advice, do you find it easy to eat the recommended foods? Does anyone prepare food for you (children, neighbours, social services)? Would you like to eat differently? Did you use to adapt your food preferences to suit others?
Difficulties	What difficulties do you experience now that you live alone, compared to before, when it comes to eating? E.g., economy, food preparation, emotions, or pleasure derived from food.
Advantages	What advantages do you experience now that you live alone, compared to before, when it comes to eating? E.g., autonomy, freedom from obligation, opportunities for innovation, or health benefits.
The social life of eating alone	Is there anything more difficult now because you have to eat alone? How do you feel about it? Do you miss how mealtimes used to be? What were mealtimes like before, and how are they now? Do you still enjoy eating? Are there any foods you’ve stopped eating due to a lack of companionship? Do you eat anything because of the memories attached to it?
Seeking commensality	Do you use TV, radio, or the internet while eating? Food and pets: Do you eat at the same time? Do you eat the same things? Exploring substitutes for food companionship. Do you meet with others to eat together? Do you share meals with others during special occasions or festivities? Do you eat at canteens? Have you ever eaten at a canteen?
Food symbolism	Do you still eat bread? Do you buy bread daily? Do you have any special meals on Sundays or weekends? Are there specific foods you eat during special occasions such as birthdays or Christmas?
Solo dining	Do you ever eat out by yourself? If so, how was the experience? Where was it, and was there a reason why? Would you do it again? What did you enjoy the most? What did you enjoy the least?

**Table 3:** Summary of the results.

Category	Subcategory	Illustrative Quote
1. Health and food ideologies in ageing	1.1. Diet as self-care and health	I eat whatever I feel like, but also what is good for my health (F73G)
	1.2. Importance of pleasure and desire	You can tell me all I need to eat, a healthy diet, that kind of diet. But the little square... the little square of chocolate I have before bed at night (F72G)
	1.3. Influence of gender	I have lost six kilos since I stopped drinking wine and alcohol (M85E)
	1.4. Influence of material conditions	Fish is very expensive: I eat frozen haddock (F73E)
2. Influence of loneliness on food choices and dietary habits	2.1. Meal simplification	I don’t get complicated with chorizo sausage or... No, on my own, I don’t do that. (F83E)
	2.2. Emergence of ready-made and prepared foods	I buy cans of cooked chickpeas or lentils. Or those cans with grilled pork (F78E)
	2.3. Food preservation strategies	What I cooked today will last me for three days. (F93E)
	2.4. Gender differences	Some nights I don’t eat anything other than an apple and a yoghurt. Or strawberries and yogurt (F83E)
3. Food consumption as a social necessity	3.1. Commensality and loss	We always used to eat together (…) Eating alone is really hard–really sad (M85E)
	3.2. Coping strategies	I put the TV on; I always put it on. Always, when I’m eating. Look, because it brings me a sense of peace (F78E)
	3.3. Routines and ritualisation	I really like them, I’ve always had [...] I still keep the same traditions as when my husband was with us (F85G)

## Data Availability

The original contributions presented in this study are included in the article. Further inquiries can be directed to the corresponding author.
